# Galectin-8 Senses Phagosomal Damage and Recruits Selective Autophagy Adapter TAX1BP1 To Control *Mycobacterium tuberculosis* Infection in Macrophages

**DOI:** 10.1128/mBio.01871-20

**Published:** 2021-07-06

**Authors:** Samantha L. Bell, Kayla L. Lopez, Jeffery S. Cox, Kristin L. Patrick, Robert O. Watson

**Affiliations:** a Department of Microbial Pathogenesis and Immunology, College of Medicine, Texas A&M Health Science Center, Bryan, Texas, USA; b Department of Molecular and Cell Biology, University of California, Berkeley, Berkeley, California, USA; Yale University School of Medicine; Max Planck Institute for Infection Biology

**Keywords:** xenophagy, bacterial pathogenesis, host-pathogen interactions, innate immunity

## Abstract

Mycobacterium tuberculosis (Mtb) causes one of the deadliest infectious diseases worldwide. Upon infection, Mtb is phagocytosed by macrophages and uses its virulence-associated ESX-1 secretion system to modulate the host cell. We showed previously that the ESX-1 secretion system perturbs the Mtb-containing phagosome, and a population (∼30%) of intracellular Mtb is tagged with ubiquitin and targeted to selective autophagy. However, our understanding of how macrophages sense and respond to damaged Mtb-containing phagosomes remains incomplete. Here, we demonstrate that several cytosolic glycan-binding proteins called galectins recognize Mtb-containing phagosomes; in macrophage cell lines and in primary macrophages, galectin-3, -8, and -9 are all recruited to the same Mtb population that colocalizes with selective autophagy markers (ubiquitin, p62, and LC3). To test whether galectins are required for controlling Mtb replication in macrophages, we generated CRISPR/Cas9 knockouts and found that galectin-8^−/−^ and galectin-3/8/9^−/−^ macrophages were similarly defective in targeting Mtb to selective autophagy and controlling replication. This suggests galectin-8 plays a unique role in anti-Mtb autophagy. In investigating galectin-8's role, we identified a novel and specific interaction between galectin-8 and the selective autophagy adapter TAX1BP1 and found that this galectin-8/TAX1BP1 interaction was necessary for macrophages to efficiently target Mtb to selective autophagy. Remarkably, overexpressing galectin-8 increased targeting of Mtb to autophagy and limited Mtb replication. Taken together, these data demonstrate that while several galectins are capable of recognizing damaged Mtb-containing phagosomes, galectin-8 plays a privileged role in recruiting downstream autophagy machinery and may represent a promising target for host-directed tuberculosis therapies.

## INTRODUCTION

Mycobacterium tuberculosis (Mtb), which causes tuberculosis (TB), infects approximately 10 million people annually and kills about 1.5 million, making it one of the deadliest infectious diseases worldwide (1). Spread in aerosolized droplets when an infected person coughs, Mtb travels to the depths of the lungs, where it is phagocytosed by alveolar macrophages. Typically, macrophages are incredibly efficient at identifying and destroying invading microbes, and they have numerous potent killing mechanisms, including lysosomal degradation, reactive oxygen species, antimicrobial peptides, guanylate-binding proteins (GBPs), and autophagy (2). However, Mtb employs strategies to resist nearly all of these defense mechanisms and survives and replicates in macrophages (3–5). Understanding the few critical mechanisms by which macrophages can successfully control Mtb is necessary for the future development of effective therapies for this difficult-to-treat pathogen.

One way a macrophage can control intracellular Mtb is through selective autophagy, a specific form of autophagy where a cell tags unwanted cytosolic cargo with ubiquitin, which serves as an “eat me” signal (6–9). Ubiquitin-tagged cargo can then be coated by a variety of selective autophagy adapters (p62/SQSTM1, CALCOCO2/NDP52, Optineurin/OPTN, etc.), which have ubiquitin-binding domains that promote their recruitment to tagged cargo. These adapters also have an LC3 interaction region (LIR), a motif that enables binding to the autophagy protein LC3 (8, 10). As a result, selective autophagy adapters serve as bridges between ubiquitinated cargo and the LC3-decorated autophagophore that will ultimately engulf the cargo before fusing with a lysosome to degrade it. Numerous types of cargo, including damaged mitochondria (mitophagy), protein aggregates (aggrephagy), and cytosolic pathogens (xenophagy), can be degraded via selective autophagy, and various subsets of adapters are associated with different types of cargo (8, 11). However, the biology underlying the redundancy and specificity of these adapters remains poorly understood.

Several lines of evidence indicate that selective autophagy is required for controlling Mtb infection. Once phagocytosed, Mtb uses its ESX-1 secretion system, which is a virulence-associated type VII secretion system, to permeabilize the Mtb phagosome. Studies have shown that ESX-1 is required for perturbing the phagosome at early time points postinfection and for readily accessing the cytosol at later time points (12–18). Our work and that of others have shown that this permeabilization allows for very early detection (within 30 min) by cytosolic sensors (13, 17, 19–21). Within 4 h of infection, approximately 30% of intracellular Mtb bacilli are surrounded by ubiquitin, LC3, and selective autophagy adapters, and in the absence of this selective autophagy targeting, Mtb survives and replicates to a higher degree (17). While the precise nature of the ubiquitination around Mtb is unclear, several E3 ligases, including Parkin, Smurf1, and TRIM16, colocalize with a subset of Mtb phagosomes and are required for optimal tagging of Mtb with ubiquitin (22–24). These E3 ligases are required for controlling Mtb replication in macrophages, and Parkin and Smurf1 are further required for controlling Mtb infection *in vivo* in mouse models of infection. Likewise, macrophages lacking the core autophagy protein ATG5 fail to control Mtb replication, and mice with a macrophage-specific ATG5 deletion are incredibly susceptible to Mtb infection and succumb within weeks (17). A subsequent report found that ATG5 plays a critical role in neutrophil-mediated inflammation, suggesting autophagy functions in both cell-intrinsic and cell-extrinsic immune responses (25).

We are continuing to understand the function, impact, and scope of selective autophagy in controlling Mtb infection, and the precise mechanisms macrophages use to detect damaged Mtb phagosomes and intracellular bacilli remain poorly defined. Our previous studies found that cytosolic DNA sensing through cGAS/STING/TBK1 is required for recognition and targeting of Mtb; macrophages lacking cGAS or STING target half as many Mtb bacilli to selective autophagy (21). However, because a sizable population of Mtb are targeted even in the absence of DNA sensing, it is likely that additional “danger signals” (e.g., microbes or damage caused by microbes) and “danger sensors” are employed by macrophages during Mtb infection (26).

One class of danger sensors are galectins, which are a large, highly conserved family of proteins that bind to glycosylated proteins and lipids via their carbohydrate recognition domains (27–29). Despite having no classical secretion signal, many galectins are extracellular where they can bind to glycans on cell surfaces or in the extracellular matrix to modulate cellular processes such as signaling, adherence, and migration (27, 28). Several galectins are also found in the cytosol where they exert other functions, including acting as soluble receptors for endosomal or lysosomal membrane damage. After disruption of membranes, galectins can access and bind to glycans within the lumen of damaged membrane-bound compartments (6, 7, 30, 31). Often, intracellular bacteria inflict endosomal damage, and galectin-3, -8, and -9 have been found to colocalize with several intracellular pathogens, including Salmonella enterica serovar Typhimurium, Shigella flexneri, Listeria monocytogenes, Legionella pneumophila, and Yersinia pseudotuberculosis (30–32). In some cases, the functional consequences of galectin recruitment to intracellular bacteria have been characterized. For example, during L. pneumophila and Y. pseudotuberculosis infection, galectin-3 promotes the recruitment of antibacterial GBPs to bacteria, and during *S.* Typhimurium infection of HeLa cells, galectin-8 recruits NDP52, which brings autophagy machinery to exposed bacteria.

Several studies have reported that galectin-3 and -8 can colocalize with Mtb and other mycobacterial species. However, to date, galectins have primarily been used as markers of membrane damage during Mtb infection, and their involvement in targeting Mtb or controlling Mtb infection are poorly understood (18, 33–38). As a result, how cell intrinsic responses, and especially selective autophagy targeting, are linked to galectin recruitment and cytosolic detection of Mtb remain open questions. Galectin-9 has been shown to enhance macrophages’ antibacterial capacity, but this cell extrinsic mechanism involves galectin-9 on the surface of macrophages binding to Tim3 on T cells to enhance macrophages’ interleukin-1β secretion (39, 40). Other groups have shown that galectin-3 and -8 colocalize with ubiquitin^+^ Mtb but did not explore the functional outcome of these colocalization events (18, 33, 35, 38). Therefore, we do not have a cohesive, systematic understanding of the mechanistic contributions of individual galectins in selective autophagy during Mtb infection, especially at early time points when initial sensing events occur.

Here, we show that galectin-3, -8, and -9 are recruited to Mtb in macrophages, and these galectin^+^ bacteria are the same population targeted to selective autophagy. Deletion of galectin-8, but not galectin-3 or -9, decreased targeting of Mtb as monitored by LC3 recruitment and by bacterial survival/replication. Deleting all three galectins did not amplify these phenotypes, suggesting galectin-8 is the most crucial for recognition and targeting of Mtb in macrophages. Using immunoprecipitation and mass spectrometry, we found that galectin-8 interacts with the selective autophagy adapter TAX1BP1, but this interaction was independent of TAX1BP1’s ubiquitin-binding domain. Furthermore, in Mtb-infected macrophages, the recruitment of TAX1BP1 to Mtb required both its interaction with galectin-8 and its ubiquitin-binding domain. Finally, we found that overexpression of galectin-8 significantly augmented macrophages’ ability to control Mtb survival and replication. This indicates galectin-8 in particular is not only essential for targeting Mtb to selective autophagy but also sufficient. This raises the possibility of targeting this detection and destruction pathway for the development of future host-directed therapies.

## RESULTS

### Galectin-3, -8, and -9 access the lumen of damaged Mtb-containing phagosomes to detect and target cytosolically exposed bacilli.

**FIG 1:**
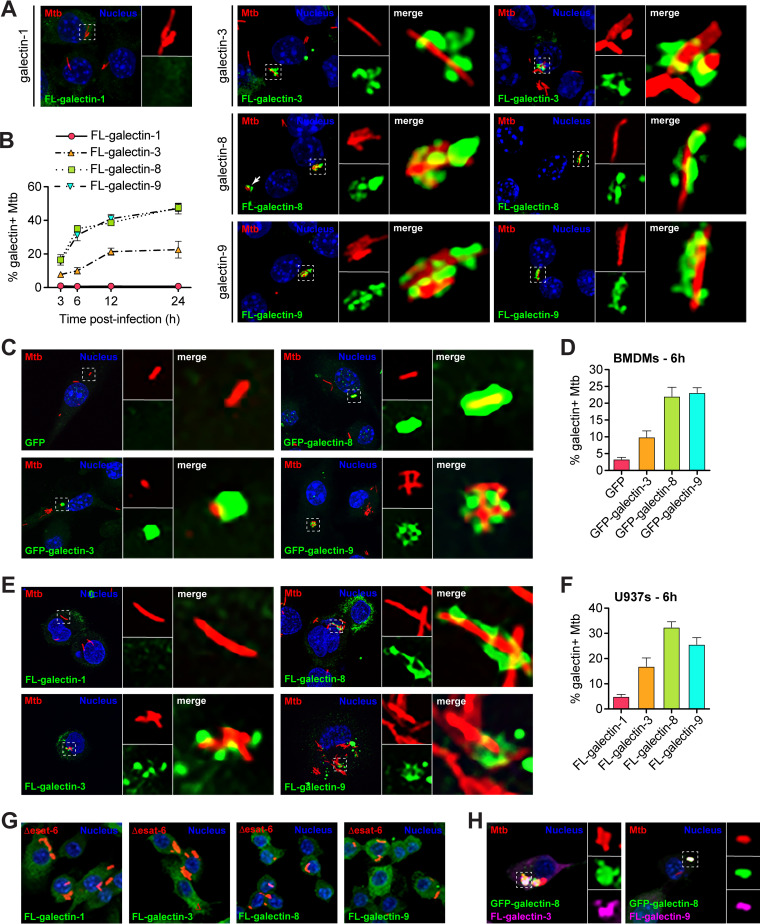
Galectins are recruited to Mtb-containing phagosomes in murine and human macrophage cell lines and in primary murine macrophages. (A) Immunofluorescence of RAW 264.7 cells stably expressing 3×FLAG (FL)-tagged galectins infected with wild-type (WT) mCherry-expressing Mtb (MOI = 1) 6 h postinfection. Green, FL-galectin; red, mCherry Mtb; blue, DAPI. (B) Quantification of FL-galectin^+^ Mtb (of indicated genotype), as shown in panel A at indicated time points. (C) Same as in panel A but in BMDMs stably expressing GFP alone or indicated GFP-tagged galectin. (D) Quantification of GFP-galectin^+^ Mtb as shown in panel C at 6 h postinfection. (E) Same as in panel A but in U937 cells. (F) Quantification of FL-galectin^+^ Mtb as shown in panel E. (G) Same as in panel A but with cells infected with Δ*esat-6* mCherry-expressing Mtb. (H) Immunofluorescence of RAW 264.7 cells stably coexpressing GFP–galectin-8 and FL-galectin-3 or FL-galectin-9 infected with WT mCherry-expressing Mtb (MOI = 1) 6 h postinfection. Green, GFP–galectin-8; magenta, FL-galectin; red, mCherry Mtb; blue, DAPI.

Because galectins have previously been implicated in sensing phagosomal damage (31, 32) and have been observed around Mtb (18, 33–36, 38), we hypothesized that they may play a critical role in sensing cytosolically exposed Mtb in macrophages. To test whether galectins were recruited to Mtb phagosomes early after infection, we generated 3×FLAG (FL)-tagged expression constructs of four different galectins: galectin-1, -3, -8, and -9. Galectin-3, -8, and -9 were chosen based on their posttranslational modifications during Mtb infection (41, 42) and because previous studies have found these galectins colocalized with intracellular pathogens (18, 31, 33–36, 38); galectin-1 was chosen as a negative control. We stably expressed epitope-tagged galectins in RAW 264.7 cells, which are murine macrophage-like cells that are a common *ex vivo* infection model for Mtb since they are genetically tractable and respond robustly to Mtb infection (21, 43). Using these cell lines (see Fig. S1A and B in the supplemental material), we infected with mCherry-expressing Mtb (Erdman strain; fully virulent) and, at various early time points postinfection (3, 6, 12, and 24 h), fixed coverslips and performed immunofluorescence microscopy to assess galectin localization relative to intracellular Mtb (Fig. 1A and B). Galectin-8 and -9 and, to a lesser extent, galectin-3 were recruited to a sizeable population of Mtb, whereas galectin-1 was not. Colocalization was detectable at 3 h postinfection and reached a maximum of ∼45% galectin-8^+^ or galectin-9^+^ bacilli after 24 h. Galectin-3 was recruited to Mtb with similar dynamics, but only to a maximum of ∼20% of bacilli after 24 h. Galectin-1 did not colocalize with Mtb at any time point examined, making it a useful negative control for future experiments.

10.1128/mBio.01871-20.1 FIG S1 Validation of galectin overexpression and knockout cell lines. (A) Western blot of RAW 264.7 cells stably expressing 3×FLAG (FL)-tagged galectins. (B and C) Levels of indicated galectin transcripts in RAW 264.7 cells stably expressing FL-galectins (B) or BMDMs stably expressing GFP-tagged galectins (C). (D) Quantification of GFP–galectin-8^+^ and FL-galectin^+^ Mtb shown in Fig. 1A. GFP–galectin-8^+^ Mtb that are also FL-galectin-3^+^ or FL-galectin-9^+^ (top) and FL-galectin-3^+^ or FL-galectin-9^+^ Mtb that are also GFP–galectin-8^+^ (bottom). Three coverslips per cell line and at least 100 bacteria per coverslip were assessed. (E to G) Levels of indicated galectin transcripts in individual galectin knockout RAW 264.7 cell lines (E), siRNA knockdown (KD) BMDMs (F), and triple galectin knockout RAW 264.7 cell lines (G). For knockout lines, error bars indicate the SEM of the averages of each of three to five clonal cell lines of each genotype. For overexpression cells and BMDM KDs, error bars indicate the SD. *, *P < *0.05; **, *P < *0.01; ***, *P < *0.005; n.s., not significant. Download FIG S1, TIF file, 2.8 MB. Copyright © 2021 Bell et al. 2021 Bell et al. https://creativecommons.org/licenses/by/4.0/ This content is distributed under the terms of the Creative Commons Attribution 4.0 International license.

To extend these findings, we next evaluated galectin recruitment to Mtb in additional macrophage types. First, we stably expressed green fluorescent protein (GFP)-tagged galectin-3, -8, or -9, or GFP only as a negative control, in primary murine macrophages (bone marrow-derived macrophages [BMDMs]) (see Fig. S1C). We infected these macrophages with mCherry Mtb and visualized galectin recruitment at 6 h postinfection. Here and in future experiments, we examined the 6 h time point since this was the earliest that we observed peak galectin recruitment to Mtb (Fig. 1B). Similar to what we observed in RAW 264.7 cells, we saw galectin-8 and -9 and, to a lesser extent, galectin-3 colocalize with Mtb (Fig. 1C and D). Next, we examined galectin recruitment in a human macrophage cell line by stably expressing 3×FLAG-tagged human galectin-3, -8, and -9 in U937 cells and using immunofluorescence to monitor colocalization 6 h postinfection. In these human macrophage cell lines, we once again observed robust recruitment of galectin-8 to Mtb, as well as considerable recruitment of galectin-3 and -9 (Fig. 1E and F). These observations across multiple macrophage types indicate that galectin recruitment is a conserved macrophage response during Mtb infection.

Next, we tested whether the ESX-1 secretion system, and therefore phagosome permeabilization, was required for galectin recruitment. To do this, we infected 3×FLAG-galectin RAW 264.7 cells with mCherry-expressing Δ*esat-6* Mtb, which is missing a key component for permeabilizing the phagosomal membrane (44). At 6 h postinfection, we did not observe colocalization of any galectin with Δ*esat-6* Mtb (Fig. 1G), indicating phagosomal permeabilization is required for galectin recruitment. Together, these findings show that the phagosomal damage, induced at least in part by ESX-1, is extensive enough to allow cytosolic proteins to access the lumen of the Mtb-containing phagosome.

We next tested whether galectin-3, -8, and -9 were all recruited to the same Mtb-containing phagosomes. To do this, we stably coexpressed GFP–galectin-8 and 3×FLAG–galectin-3 or -9 in RAW 264.7 cells and again infected them with mCherry Mtb. We found that galectin-8 and -9 colocalized in almost all instances (Fig. 1H; see also Fig. S1D). Likewise, galectin-3 was present on almost all galectin-8^+^ Mtb, but a large portion of galectin-8^+^ Mtb did not have galectin-3 present (Fig. 1H; see also Fig. S1D). This suggests that the same population of ∼30% of intracellular Mtb accumulates galectin-8 and -9 and sometimes galectin-3.

**FIG 2:**
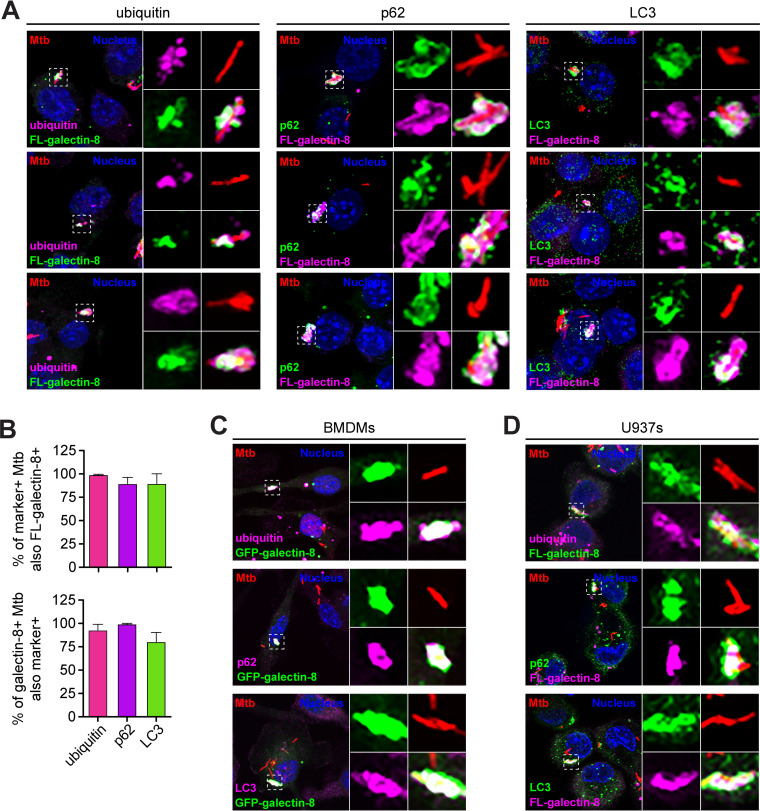
Galectin-decorated Mtb-containing phagosomes colocalize with selective autophagy markers. (A) Immunofluorescence of RAW 264.7 cells stably expressing 3×FLAG (FL)-tagged galectin-8 infected with WT mCherry Mtb (MOI = 1) 6 h postinfection costained for indicated selective autophagy marker (ubiquitin, p62, and LC3). Green and magenta, endogenous selective autophagy marker or FL-galectin-8 (as indicated); red, mCherry Mtb; blue, DAPI. (B) Quantification of marker-positive and FL-galectin-8^+^ Mtb bacilli shown in panel A. Marker-positive Mtb that are also FL-galectin-8^+^ (top) and FL-galectin-8^+^ Mtb that are also marker-positive (bottom) are shown. Error bars indicate the SD of three coverslips per marker with at least 100 bacteria assessed. (C and D) Same as in panel A but in BMDMs expressing GFP–galectin-8 (C) or in U937 cells expressing FL-galectin-8 (D).

Based on previous reports and the size of the galectin^+^ Mtb population, we hypothesized that galectin^+^ Mtb-containing phagosomes would be positive for selective autophagy markers (18, 33, 35, 38). To test this, we costained RAW 264.7 cells for 3×FLAG–galectin-8 and a panel of selective autophagy markers, including ubiquitin (the “eat me” signal), p62 (a selective autophagy adapter), and LC3 (the autophagosome marker). As predicted, a vast majority of the galectin-8^+^ Mtb were also positive for ubiquitin, p62, and LC3 at 6 h postinfection (Fig. 2A and B). The reverse was also true: the ubiquitin^+^, p62^+^, and LC3^+^ Mtb strains were nearly all also galectin-8^+^ (Fig. 2B). We observed the same colocalization between galectin-8 and selective autophagy markers in both BMDMs and U937 cells (Fig. 2C and D). This indicates that galectin^+^ Mtb are indeed the same population of Mtb that are targeted to selective autophagy.

### Loss of galectin-8 decreases targeting of Mtb to selective autophagy.

**FIG 3:**
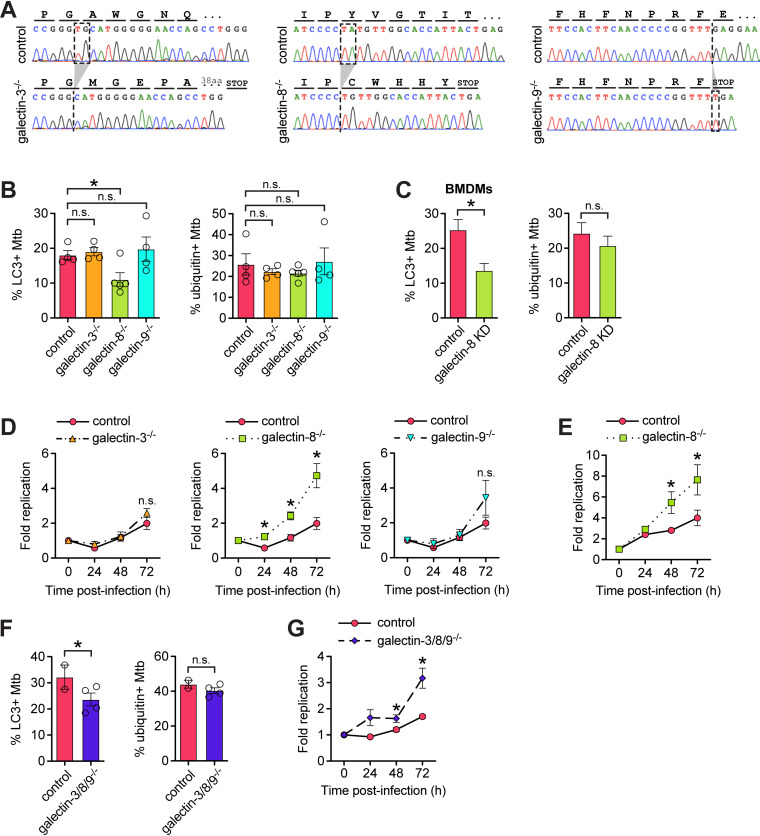
Galectin-8 is required to efficiently target Mtb to selective autophagy to control Mtb replication in macrophages. (A) Representative chromatograms from galectin-3, -8, and -9 knockout cells lines indicating the nature of nonsense mutations introduced via CRISPR/Cas9. (B) Quantification of LC3- and ubiquitin-positive Mtb in control (sgRNAs targeting GFP) or individual galectin knockout RAW 264.7 cell lines 6 h postinfection. Circles represent data for each clonally selected cell line. (C) Same as in panel B but in BMDMs with galectin-8 knocked down via siRNA. (D) Fold replication of luxBCADE Mtb (MOI = 1) in control and knockout RAW 264.7 cells at the indicated time points. Data normalized to *t* = 0 h and representative of at least three independent experiments. (E) Same as in panel D but with replication measured by enumerating CFU. (F and G) Same as in panels B and D but with RAW 264.7 cell lines in which all three galectins are knocked out via CRISPR/Cas9. Error bars indicate the SEM of knockout cell lines or the SD for knockdown cells; for immunofluorescence (IF), at least 300 bacteria per cell line were assessed. *, *P < *0.05; n.s., not significant.

We next sought to determine whether recruitment of galectins is required for targeting Mtb to antibacterial selective autophagy. To do this, we used a lentiviral CRISPR/Cas9 system to mutate the genes encoding galectin-3, -8, or -9 (*Lgals3*, *Lgals8*, and *Lgals9*) in RAW 264.7 cells. We designed small guide RNAs (sgRNAs) targeting the first one to two coding exons of each galectin gene; we used GFP-targeted sgRNAs as negative controls. After transducing RAW 264.7 cells stably expressing FLAG-Cas9 with lentiviral sgRNAs constructs, we antibiotic-selected cells, isolated clonal populations, and validated homozygous mutation by sequencing the targeted region. We chose clonal populations that had one or two base pair insertions or deletions that resulted in frameshift mutations early in the transcript (exon 1 or 2) (Fig. 3A). To limit the possibility of off-target and bottleneck effects, we used at least three clonal populations for each gene, and these were derived from two different gRNAs per gene. Since we were unable to identify commercial antibodies that reliably detected the three mouse galectins, we further validated loss of gene expression in the knockout cell lines using RT-qPCR since the mutated transcripts should be degraded via nonsense mediated decay. As expected, all of the knockout cell lines had significantly diminished mRNA expression of the sgRNA-targeted galectin (see Fig. S1E).

Next, we tested whether these knockout cell lines could efficiently target Mtb to selective autophagy. We infected with mCherry Mtb, stained for the autophagy marker LC3, and quantified the percentage of targeted bacteria. Compared to control cell lines (GFP sgRNAs), galectin-8^−/−^ cell lines had less (approximately 50% less) LC3^+^ bacteria at 6 h postinfection (Fig. 3B). This was specific to galectin-8 since galectin-3^−/−^ and galectin-9^−/−^ cell lines had similar percentages of LC3^+^ Mtb compared to controls. These cell lines all had similar proportions of ubiquitin^+^ Mtb (Fig. 3B), which suggests that galectin recruitment is independent of ubiquitination. To also test this in primary macrophages, we knocked down galectin-8 in BMDMs using siRNA transfection (see Fig. S1F), and similarly found that in macrophages deficient in galectin-8, there were fewer LC3^+^ Mtb but similar numbers of ubiquitin^+^ Mtb compared to controls (Fig. 3C).

To test how this defect in targeting would impact Mtb survival/replication in macrophages, we measured bacterial replication using an Mtb strain constitutively expressing luxBCADE. With this strain, at various time points postinfection, we can use luminescence as a proxy to quickly and easily monitor Mtb replication in numerous control and knockout RAW 264.7 cell lines (41–43). In wild-type cells, Mtb replication was well controlled; bacterial burdens decreased after 24 h before Mtb began to slowly replicate intracellularly at later time points (Fig. 3D). However, in galectin-8^−/−^ macrophages, but not in galectin-3^−/−^ or galectin-9^−/−^ macrophages, Mtb was not controlled at 24 h postinfection, and bacterial burdens were significantly higher throughout the course of infection (Fig. 3D). To confirm this result, we also measured bacterial replication/survival using colony-forming units (CFUs) and again found that galectin-8^−/−^ macrophages had higher bacterial burdens compared to controls (Fig. 3E). These data indicate that the defective selective autophagy targeting in galectin-8^−/−^ macrophages results in diminished control of Mtb survival/replication, and galectin-8 in particular is required for targeting Mtb to antibacterial selective autophagy.

Because several galectins are recruited to Mtb, we next investigated whether they served redundant functions in targeting Mtb to selective autophagy. We adapted a lentiviral sgRNA array construct to simultaneously express the most efficient galectin-specific sgRNAs (or GFP sgRNAs as a negative control) in FLAG-Cas9-expressing RAW 264.7 cells. As with the single-knockout lines, we isolated clonal populations, confirmed homozygous mutation of all three galectin genes, and validated the triple-knockout cell lines by measuring galectin transcript levels (see Fig. S1G). We infected the galectin-3/8/9^−/−^ triple-knockout cells and GFP sgRNA control cells with mCherry Mtb and used immunofluorescence microscopy to quantify selective autophagy targeting. Compared to controls, the galectin-3/8/9^−/−^ cell lines had fewer LC3^+^ Mtb 6 h postinfection, but similar numbers of ubiquitin^+^ Mtb (Fig. 3F). When infected with luxBCADE Mtb, the galectin-3/8/9^−/−^ cell lines also had higher Mtb survival/replication compared to controls (Fig. 3G). Surprisingly, the magnitude of the defect in the galectin-3/8/9^−/−^ triple-knockout cells essentially phenocopied that of the galectin-8^−/−^ single-knockout cells, suggesting these three galectins do not serve redundant functions, and galectin-8 in fact has a privileged role in targeting Mtb to selective autophagy.

### Galectin-8 interacts with diverse proteins involved in exosome secretion, membrane trafficking, and selective autophagy.

**FIG 4:**
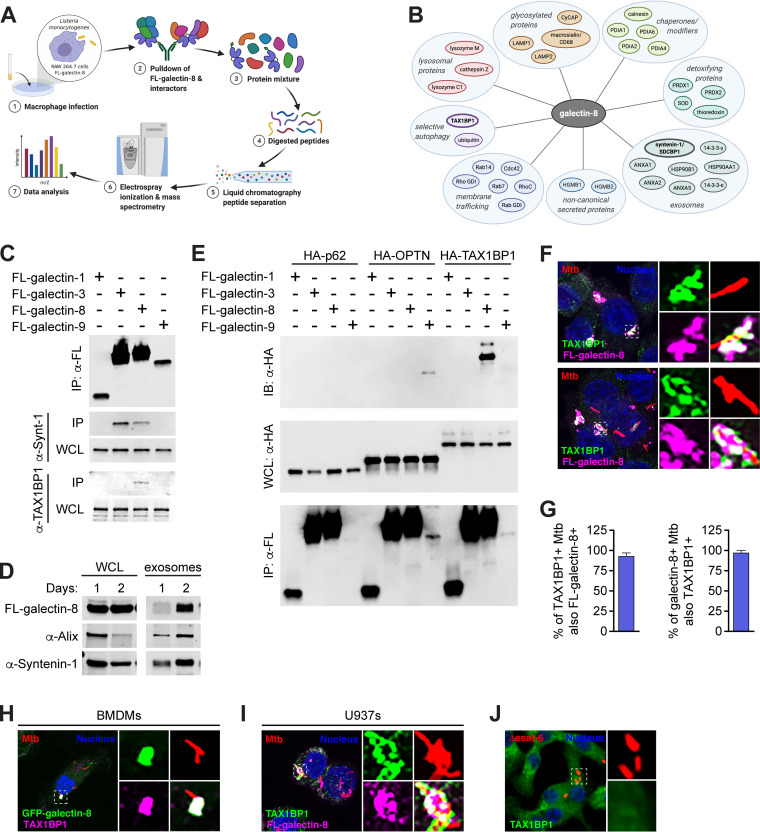
Galectin-8 interacts with exosome-associated proteins and selective autophagy adapter TAX1BP1. (A) Schematic of experimental approach for immunoprecipitation and mass spectrometry (IP-LC/MS) identification of galectin-8 binding partners in macrophages during intracellular bacterial infection. (B) Proteins identified by IP-LC/MS as interacting with galectin-8. (C) Coimmunoprecipitation (IP) of 3×FLAG (FL)-tagged galectins ectopically expressed in HEK293T cells. Whole-cell lysates (WCL) and co-IPs probed for endogenous syntenin-1 and TAX1BP1. (D) WCL and exosomes from FL-galectin-8-expressing RAW 264.7 cells cultured for indicated number of days to assess exosomes accumulated in cell culture media. Endogenous Alix and syntenin-1 were used as markers for exosomes. (E) Directed co-IPs of FL-galectins and HA-tagged selective autophagy adapters expressed ectopically in HEK293T cells. (F) Immunofluorescence of RAW 264.7 cells stably expressing FL-galectin-8 and costained for endogenous TAX1BP1 infected with WT mCherry-expressing Mtb (MOI = 1) 6 h postinfection. Green, endogenous TAX1BP1; magenta, FL-galectin-8; red, mCherry Mtb; blue, DAPI. (G) Quantification of galectin-8^+^ and TAX1BP1^+^ Mtb shown in panel F. (H) Same as in panel F but in BMDMs stably expressing GFP–galectin-8. Green, GFP–galectin-8; magenta, endogenous TAX1BP1; red, mCherry Mtb; blue, DAPI. (I) Same as in panel F but in U937s. (J) Immunofluorescence of RAW 264.7 cells infected with Δ*esat-6* mCherry Mtb and stained for endogenous TAX1BP1 at 6 h postinfection. Green, endogenous TAX1BP1; red, Δ*esat-6* Mtb; blue, DAPI. Error bars indicate the SD of three coverslips with at least 100 bacteria assessed.

To gain a deeper understanding of how galectin-8 promotes targeting of Mtb to selective autophagy, we used an unbiased mass spectrometry approach. We predicted that galectin-8 may have one or more specific binding partners that would help explain why loss of galectin-8 in particular decreased LC3 recruitment to Mtb-containing phagosomes. Due to technical limitations resulting from Mtb’s classification as a Biosafety Level 3 (BSL3) pathogen, we turned to Listeria monocytogenes, a BSL2 pathogen that also elicits a type I IFN response, can be targeted to selective autophagy, and recruits galectin-3, -8, and -9 (13, 31, 45). To increase the population of L. monocytogenes targeted to selective autophagy, we used a strain lacking ActA, a protein that enables mobility within the host cell and therefore helps bacteria evade autophagy (45). We infected RAW 264.7 cells stably expressing 3×FLAG–galectin-8 with Δ*actA* L. monocytogenes and immunoprecipitated (IP) galectin-8. Proteins associated with galectin-8 were then identified by using liquid chromatography-mass spectrometry (LC/MS) (Fig. 4A).

The interacting partners identified by IP-LC/MS provided insight into several novel aspects of galectin-8 biology (Fig. 4B). First, consistent with galectin-8 recognizing damaged phagosomes, endosomes, and lysosomes, we found lysosomal proteins (cathepsin Z, lysozyme M, and lysozyme C1), highly glycosylated proteins (LAMP1, LAMP2, macrosialin/CD68, and cyclophilin C-associated protein), chaperones/modifiers of glycosylated proteins (calnexin and protein disulfide isomerases [PDIA1, PDIA3, PDIA4, and PDIA6]), and detoxifying enzymes (thioredoxin, superoxide dismutase, and peroxireductases [PRDX1 and PRDX2]). In addition, we identified several galectin-8 binding partners with known roles in membrane trafficking (Rab7, Rab14, RhoC, Cdc42, Rab GDI [GDP dissociation inhibitor], and Rho GDI) and cytoskeleton rearrangements (EFhd2, profilin, talin-1, gelsolin, F-actin capping proteins, and macrophage capping protein), which are all consistent with galectin-8’s role in recognizing damaged endosomes and lysosomes.

Interestingly, we identified several proteins that, like galectins, are secreted through a noncanonical pathway that does not require a signal sequence, including HGMB1 (46, 47). Also identified were a panel of proteins associated with exosome secretion, a form of noncanonical secretion, including syntenin-1/SDCBP, HSP90AA1, HSP90B1, ANXA1, ANXA2, ANXA5, 14-3-3-epsilon/YWAHAE, and 14-3-3-gamma/YWAHAG (48–51). Using coimmunoprecipitations (co-IPs) of 3×FLAG-tagged galectins ectopically expressed in HEK293T cells, we confirmed this interaction between galectin-8 and endogenous syntenin-1 (Fig. 4C). This interaction was not unique to galectin-8, since galectin-3, but not galectin-1 or -9, also interacted with syntenin-1. These observations led us to hypothesize that galectin-8 could be secreted via exosomes. To test this, we isolated exosomes from the cell culture supernatant of RAW 264.7 cells and found that 3×FLAG–galectin-8, along with the exosomal proteins Alix and syntenin-1, were present in exosome preps (Fig. 4D). Moreover, the amount of exosomal galectin-8, Alix, and syntenin-1 increased over time as exosomes accumulated in the cell culture media. Together, these data suggest that release in exosomes may be a key mechanism of secretion for extracellular galectins.

Finally, our mass spectrometry analysis identified ubiquitin, which is consistent with our observation that galectin-8 colocalizes with ubiquitin^+^ Mtb (Fig. 2), and it corroborates recent studies using global proteomics approaches that found galectin-8 itself is ubiquitinated during Mtb infection (41, 42). We also identified TAX1BP1 as a galectin-8 binding partner in our IP-LC/MS. While TAXBP1 has been previously characterized as a selective autophagy adapter with ubiquitin- and LC3-binding domains, it is not known to interact with galectins. We speculated that galectin-8 could augment selective autophagy of Mtb by binding to TAX1BP1 and promoting recruitment of downstream autophagy machinery.

### Galectin-8 interacts with TAX1BP1 independently of ubiquitination.

We first confirmed the galectin-8/TAX1BP1 interaction using HEK293T cells ectopically expressing 3×FLAG-galectins and found that endogenous TAX1BP1 immunoprecipitated specifically with galectin-8 (Fig. 4C). To further probe the specificity of the galectin-8/TAX1BP1 interaction, we generated hemagglutinin (HA)-tagged expression constructs for several selective autophagy adapters, including TAX1BP1, p62, and optineurin/OPTN. We then tested the interaction between each galectin and adapter by coexpressing pairs in HEK293T cells and performing directed co-IPs. Remarkably, we found that galectin-8 specifically interacted with TAX1BP1 and no other adapters, and TAX1BP1 interacted specifically with galectin-8 and no other galectins (Fig. 4E). We also detected an unexpected but seemingly specific interaction between galectin-9 and OPTN (Fig. 4E). These highly specific protein-protein interactions are surprising since there is a high degree of similarity between galectins (84 to 97% similarity; see Fig. S2A and B in the supplemental material).

10.1128/mBio.01871-20.2 FIG S2 Domains of galectins and adapter proteins. (A) Schematic of galectin-1, -3, -8, and -9 domains. CRD, carbohydrate recognition domain. (B) Percent conservation in pairwise comparisons of CRDs by analysis with M-Coffee. (C) Domain structure of selective autophagy adapters used in this study. SKICH, coiled-coil, and PB1 are protein-protein interaction domains. CLIR, noncanonical/LC3C-interacting region; LIR, LC3-interacting region; ZF, zinc finger; UB, ubiquitin-recognition domain; UBZ, ubiquitin-binding zinc finger domain. Download FIG S2, TIF file, 2.3 MB. Copyright © 2021 Bell et al. 2021 Bell et al. https://creativecommons.org/licenses/by/4.0/ This content is distributed under the terms of the Creative Commons Attribution 4.0 International license.

We next examined the localization of TAX1BP1 during Mtb infection. We infected RAW 264.7 cells expressing 3×FLAG–galectin-8 with mCherry Mtb and used immunofluorescence microscopy to visualize endogenous TAX1BP1. TAX1BP1 colocalized with galectin-8^+^ Mtb (Fig. 4F), and we found near complete overlap in the TAX1BP1^+^ and galectin-8^+^ populations (Fig. 4G). TAX1BP1 was also recruited to Mtb in both BMDMs and U937s and also colocalized with galectin-8 in these macrophage types (Fig. 4H and I). In cells infected with Δ*esat-6* Mtb, TAX1BP1 did not colocalize with Mtb (Fig. 4J). This indicates that, like galectins (Fig. 1C) and other adapters (17), phagosomal damage and/or cytosolic exposure is required for the recruitment of TAX1BP1.

Because we detected an interaction between galectin-9 and OPTN, we performed similar experiments costaining for OPTN. However, we found that endogenous OPTN did not colocalize with Mtb (see Fig. S2C). However, when we stably expressed 3×FLAG-OPTN in RAW 264.7 cells, we observed low levels of colocalization (see Fig. S2C), suggesting that while OPTN is capable of being recruited to the Mtb-containing phagosome, it likely does not play a substantial role in the early targeting of Mtb to selective autophagy under normal conditions. Because several galectins (galectin-3, -8, and -9) and selective autophagy adapters (TAX1BP1 and p62) are all recruited to the same population of Mtb-containing phagosomes, the highly specific galectin-8/TAX1BP1 interaction is particularly noteworthy.

**FIG 5:**
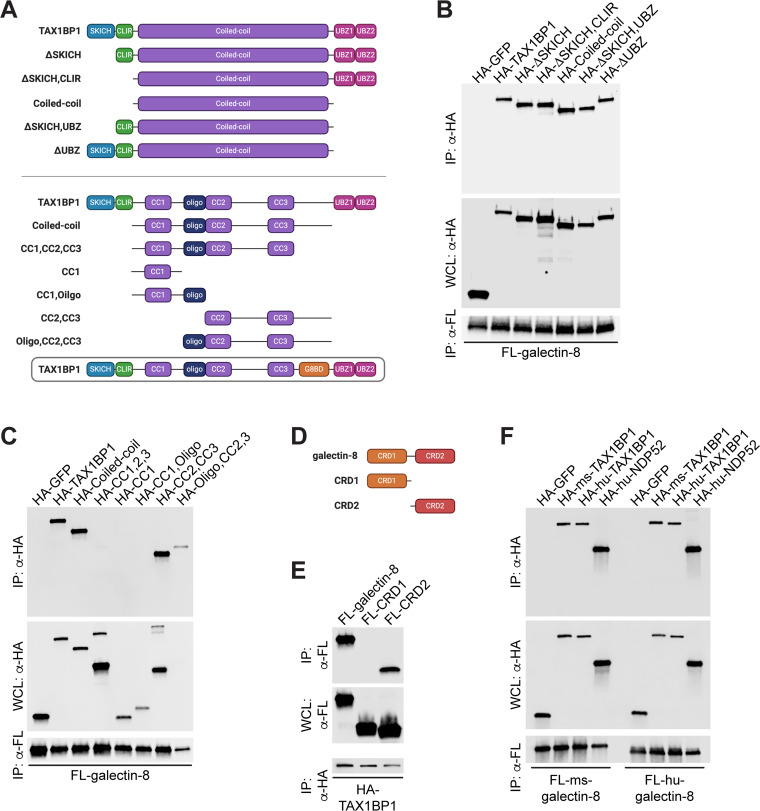
TAX1BP1’s coiled-coil domain and galectin-8’s CRD2 are required for their interaction. (A) Schematic representation of TAX1BP1 domain structure and truncations used in panels B and C. CLIR, noncanonical/LC3C-interacting region; UBZ, ubiquitin-binding zinc finger domain; CC, coiled-coil domains; oligo, oligomerization domain. (B and C) Directed coimmunoprecipitations (IP) of 3×FLAG (FL)-tagged galectin-8 ectopically expressed in HEK293Ts. Whole-cell lysates (WCL) and co-IPs probed for HA-tagged TAX1BP1 truncations. HA-GFP shown as negative control for interaction. (D) Schematic of galectin-8 domain structure and truncations. CRD, carbohydrate recognition domain. (E) Directed co-IPs of HA-TAX1BP1 expressed in HEK293T cells. WCLs and co-IPs probed for FL-galectin-8 truncations. (F) Same as in panels B and C but with mouse (ms) and human (hu) FL-galectin-8, HA-TAX1BP1, and HA-NDP52.

To investigate further how these proteins interact, we made a series of truncations of both galectin-8 and TAX1BP1. TAX1BP1 contains several annotated domains, including a SKICH domain, an LIR, a large coiled-coil domain, and two ubiquitin-binding zinc fingers (UBZs) (Fig. 5A). Because galectin-8 itself is likely ubiquitinated during infection, we predicted that TAX1BP1 would bind to galectin-8 via its UBZ domains. Surprisingly, when we performed directed co-IPs between galectin-8 and a panel of TAX1BP1 truncations, we found that the UBZ domains of TAX1BP1 were dispensable for its interaction with galectin-8 in this system (Fig. 5B). Instead, only the coiled-coil domain was required for interaction. To further narrow the region required for interaction with galectin-8, we tested additional truncations of TAX1BP1 that included combinations of the N and C termini of the coiled-coil domain, an annotated oligomerization domain, and three smaller coiled-coil domains (Fig. 5A). In co-IPs with galectin-8 and these additional TAX1BP1 truncations, we found that the C-terminal portion of the coiled-coil domain was required and sufficient for this interaction (Fig. 5C). We propose calling this region of TAX1BP1 the galectin-8-binding domain (G8BD) (Fig. 5A). We next investigated truncations of galectin-8, which contains two carbohydrate recognition domains (CRDs) that are connected by a short flexible linker (Fig. 5D). In directed co-IPs, we found that the C-terminal CRD domain (CRD2), but not the N-terminal CRD (CRD1), interacted with TAX1BP1 (Fig. 4E). Together, these biochemical experiments indicate that TAX1BP1 has evolved a ubiquitin-independent mechanism to specifically interact with galectin-8.

A previous study found that in nonimmune cells, galectin-8 interacts with another selective autophagy adapter, NDP52, which has a domain structure highly similar to TAX1BP1 (see Fig. S2D) (31, 52). This study found that, similar to our findings with TAX1BP1, human NDP52 interacts with galectin-8 via the C-terminal region of NDP52’s comparatively smaller coiled-coil domain. Because of these similarities, we wanted to test the conservation of the TAX1BP1/galectin-8 interaction. To do this, we coexpressed human 3×FLAG–galectin-8 with human HA-TAX1BP1 or human HA-NDP52 and performed co-IPs. Consistent with previous reports, galectin-8 interacted with NDP52 (Fig. 5F). Importantly, human galectin-8 also interacted with human TAX1BP1 (Fig. 5F). This previously unidentified interaction indicates that galectin-8 can interact with both NDP52 and TAX1BP1 in human cells. Based on our previous studies, the mouse gene encoding NDP52 appears to be disrupted by repetitive elements and lacks the regions previously shown to interact with galectin-8. Therefore, while the reported interaction between NDP52 and galectin-8 is likely not at play in mouse cells, it appears that human cells have evolved galectin-8 binding partners that may serve redundant functions. Finally, mouse galectin-8 can interact with human TAX1BP1 and human NDP52, and human galectin-8 can interact with mouse TAX1BP1 (Fig. 5F), which suggests that the biochemical interactions of galectin-8 and TAX1BP1 are highly conserved.

### TAX1BP1 can be recruited to Mtb-containing phagosomes by binding galectin-8 or ubiquitinated proteins.

**FIG 6:**
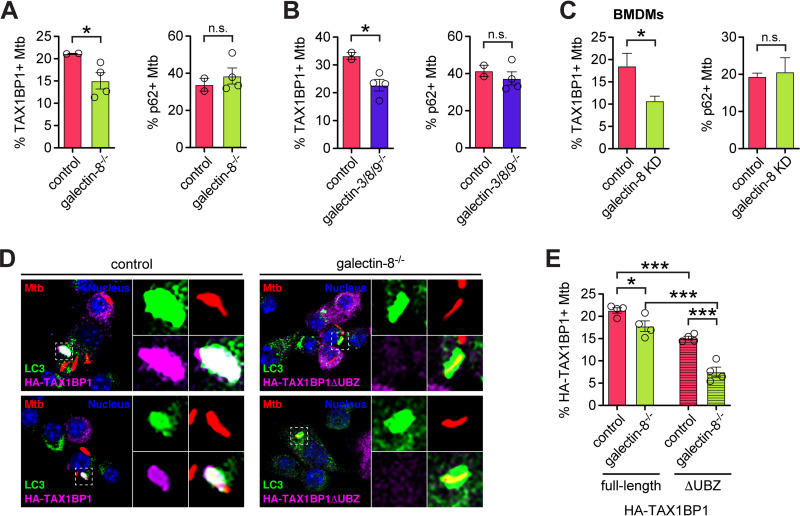
TAX1BP1 is recruited to Mtb-containing phagosomes by both its UBZ domain and its interaction with galectin-8. (A) Quantification of TAX1BP1^+^ (left) or p62^+^ (right) Mtb in control galectin-8 knockout RAW 264.7 cell lines at 6 h postinfection. Circles represent data for individual clonally selected cell lines. (B and C) Same as in panel A but in RAW 264.7 cell lines in which all three galectins are knocked out (B) or galectin-8 knockdown (KD) BMDMs (C). (D) Immunofluorescence of control or galectin-8 knockout RAW 264.7 cells stably expressing full-length HA-TAX1BP1 or truncated HA-TAX1BP1ΔUBZ. Cells were infected with WT mCherry-expressing Mtb (MOI = 1) and harvested at 6 h postinfection. Green, LC3; magenta, HA-TAX1BP1 variants; red, mCherry Mtb; blue, DAPI. (E) Quantification of indicated variant HA-TAX1BP1-positive Mtb in indicated genotype. Error bars indicate the SEM of knockout cell lines or the SD for knockdown cells; at least 300 bacteria per cell line were assessed. *, *P < *0.05; ***, *P < *0.005; n.s., not significant.

To assess how the galectin-8/TAX1BP1 interaction influences targeting of Mtb to selective autophagy, we looked at the recruitment of TAX1BP1 to Mtb in galectin-8-deficient cells. The percentages of TAX1BP1^+^ Mtb in both galectin-8^−/−^ and galectin-3/8/9^−/−^ RAW 264.7 cells were lower compared to controls (Fig. 6A and B). Likewise, in galectin-8 knockdown BMDMs, fewer Mtb bacilli were TAX1BP1^+^ (Fig. 6C). This defect in recruitment was specific to TAX1BP1 since in all of these cells, the number of p62^+^ Mtb was similar in controls and galectin-8-deficient cells (Fig. 6A to C).

Because a sizeable population of Mtb was TAX1BP1^+^ even in the absence of galectin-8, we next investigated how specific domains of TAX1BP1 might contribute to its colocalization with Mtb. We predicted that because TAX1BP1 has UBZ domains, perhaps it could be recruited to Mtb in the absence of galectin-8 by binding to other ubiquitinated substrates surrounding the Mtb-containing phagosome. To test this, we stably expressed full-length HA-TAX1BP1 or HA-TAX1BP1 lacking the UBZ domains (HA-TAX1BP1ΔUBZ) in control and galectin-8^−/−^ cell lines. At 6 h postinfection with mCherry Mtb, we performed immunofluorescence microscopy to quantify the number of HA-TAX1BP1^+^ bacteria (Fig. 6D). Consistent with the experiments in Fig. 6A, which examined endogenous TAX1BP1, full-length HA-TAX1BP1 was recruited less efficiently in galectin-8^−/−^ cells (Fig. 6E), again indicating the galectin-8/TAX1BP1 interaction is required for TAX1BP1 recruitment. Furthermore, in control cells expressing HA-TAX1BP1ΔUBZ, even fewer Mtb were TAX1BP1^+^ (Fig. 6E), suggesting that TAX1BP1’s ability to bind ubiquitinated substrates is also required for its recruitment to Mtb. Finally, in support of our prediction, HA-TAX1BP1ΔUBZ was recruited least efficiently in galectin-8^−/−^ cells (Fig. 6E), suggesting that both binding capabilities are involved in recruiting TAX1BP1 to Mtb. The residual recruitment of TAX1BP1 to Mtb in the absence of both galectin-8 and UBZ domains could be mediated by TAX1BP1’s LIR or other domains or its oligomerization with endogenous wild-type TAX1BP1. Together, these data demonstrate that TAX1BP1 can be recruited to damaged Mtb-containing phagosomes by at least two independent mechanisms: binding to galectin-8 via its coiled-coil domain (the G8BD) and binding to ubiquitinated substrates.

### Overexpression of galectin-8 augments targeting to selective autophagy.

**FIG 7:**
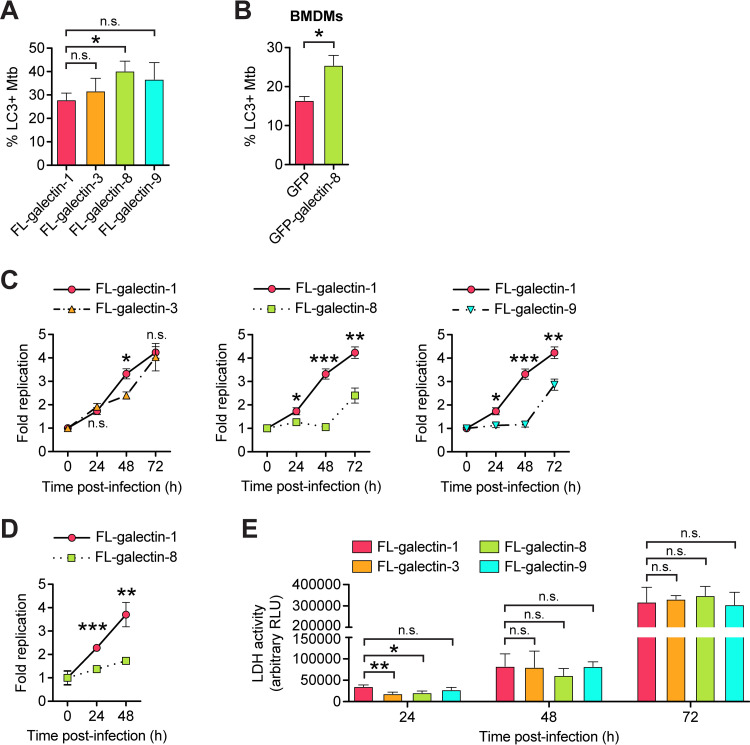
Overexpression of galectin-8 increases targeting of Mtb and controls Mtb replication. (A) Quantification of LC3^+^ Mtb in RAW 264.7 cells overexpressing 3×FLAG (FL)-tagged galectins at 6 h postinfection. (B) Same as in panel A but for BMDMs stably expressing GFP-tagged galectin-8. (C) Fold replication of luxBCADE Mtb (MOI = 1) in FL-galectin overexpression RAW 264.7 cells at the indicated time points. Data are normalized to *t* = 0 h and representative of at least three independent experiments. (D) Same as in panel C but with the fold replication measured by enumerating CFU. (E) LDH release as measured by an LDH-Glo cytotoxicity assay in FL-galectin RAW 264.7 cells. Culture supernatants were collected and assayed at indicated times postinfection. Error bars indicate the SD; for IF, at least three coverslips per cell line and 100 bacteria per coverslip were assessed. *, *P < *0.05; **, *P < *0.01; ***, *P < *0.005; n.s., not significant.

Finally, having characterized the requirement of galectins for targeting Mtb to selective autophagy, we next tested how overexpression of galectins might impact this pathway. RAW 264.7 cells overexpressing 3×FLAG–galectin-8 had a small but significant and reproducible increase in LC3^+^ Mtb at 6 h postinfection compared to cells overexpressing FL-galectin-1 (Fig. 7A). Similarly, BMDMs overexpressing GFP–galectin-8 also had an increase in LC3^+^ Mtb compared to BMDMs expressing GFP alone (Fig. 7B). Importantly, the increased targeting in FL-galectin-8 RAW 264.7 cells translated to a significant increase in macrophages’ ability to control Mtb replication as measured by both luxBCADE Mtb and CFU (Fig. 7C and D). Overexpression of FL-galectin-9 also augmented cells’ ability to control Mtb replication (Fig. 7C), but our data suggest that this is independent of selective autophagy targeting (Fig. 7A). Importantly, the decrease in Mtb survival/replication in these cells is not due to cell death of FL-galectin-8 or FL-galectin-9 overexpression cells; lactate dehydrogenase (LDH) release, a readout of cell death, increased in all cell lines over the course of a 72 h infection, but there was not increased cell death in FL-galectin-8 or FL-galectin-9 cells compared to controls (Fig. 7E). Therefore, it seems that the decrease in Mtb survival/replication in FL-galectin-8 cells is attributable to them more efficiently targeting Mtb to selective autophagy. Together, these data indicate that overexpression of galectin-8 substantially enhances macrophages’ ability to detect and control Mtb infection.

## DISCUSSION

**FIG 8:**
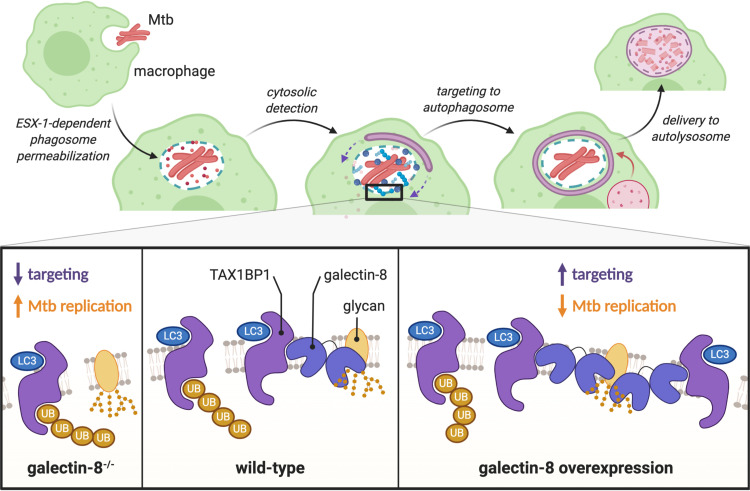
Galectin-8 and TAX1BP1 recognize and target Mtb to selective autophagy in macrophages. Schematic representation of how Mtb is detected by macrophages. Galectin-8 binds to cytosolically exposed glycans in the lumen of damaged Mtb-containing phagosomes. TAX1BP1 is recruited to these damaged phagosomes via its interaction with galectin-8, as well as through its interaction with ubiquitinated substrates. Deletion of galectin-8 results in less targeting of Mtb and increased Mtb survival/replication, while overexpression of galectin-8 leads to increased targeting and less Mtb replication.

Selective autophagy is a critical pathway employed by macrophages to control Mtb infection. Here, we characterized the involvement of galectins, a family of damage/danger sensors, in the selective autophagy response to Mtb (Fig. 8). Of the galectins we studied, we found that galectin-8, but not galectin-3 or -9, was required for controlling Mtb infection in macrophages. This is somewhat surprising since all three galectins were recruited to the phagosome. However, the specific requirement of galectin-8 seems to be due to its highly specific interaction with the adapter TAX1BP1, which a recent report found to be required for targeting Mtb to selective autophagy and controlling Mtb replication in macrophages (41). Our data indicate that TAX1BP1 can be recruited to the Mtb-containing phagosome in two ways: by binding directly to galectin-8, which is recruited directly to damaged Mtb-containing phagosomes, and by binding to ubiquitinated substrates. Consistently, this two-pronged recruitment of an adapter, via galectin-8 and via ubiquitinated substrates, has also been observed for NDP52 in HeLa cells infected with *S.* Typhimurium (31). Since NDP52 and TAX1BP1 are highly related selective autophagy adapters, it is perhaps not surprising that they have similar functional profiles. The curious similarities and apparent redundancies between adapters emphasize the importance of understanding the nature of their specific biological functions. Many important questions remain to be explored, including whether TAX1BP1 and NDP52 serve truly redundant roles in human autophagy or if they have evolved cell-type specific functions or particularly unique responsibilities, such as interacting with motor proteins to promote cargo trafficking (53, 54).

Our experiments demonstrate that even early during infection, when Mtb appears to be enclosed inside a vacuole, there is sufficient disruption of the phagosomal membrane to permit entry of host factors into the lumen of the Mtb-containing phagosome. As a result, there is likely substantial exposure of both pathogen-associated and damage-associated molecular patterns (PAMPs and DAMPs) very early during Mtb infection. Some of the host pattern recognition receptors that detect these danger signals are known, including cGAS and now, galectins. These studies indicate that the molecular environment around the Mtb-containing phagosomes is extremely complex and crowded with many proteins involved in various host response pathways: cGAS (STING/TBK1/TRIM14/IRF3), galectins, and ubiquitin (adapters [p62, TAX1BP1, NDP52, and NBR1], LC3s/GABARAPs, E3 ubiquitin ligases [Parkin, TRIM16, and Smurf]). However, the mechanistic links between these different proteins and pathways remain somewhat obscure. As a kinase, TBK1 can phosphorylate adapters such as OPTN, NDP52, and p62 (55, 56), and phosphorylation of OPTN by TBK1 can increase its affinity for ubiquitin, but it remains unknown whether TBK1 activation influences adapters’ affinity for ubiquitin, LC3, or galectins during Mtb infection. Furthermore, the E3 ubiquitin ligases Parkin, Smurf1, and TRIM16 colocalize with Mtb and contribute to the ubiquitin cloud surrounding bacteria (22–24), but how these E3 ligases are activated and recruited upon Mtb infection and what proteins each E3 modifies are lingering unanswered questions. Since Mtb is an exquisitely evolved pathogen, it is very likely that yet-to-be-identified bacterial proteins are intimately involved in these processes. The recent discovery of a ubiquitin-binding protein (Rv1468c) on Mtb’s surface and previous reports of Mtb interfering with autophagy regulation suggest that Mtb does indeed have mechanisms for modulating the host’s selective autophagy pathway (57–59).

In our studies, we found that galectin-8 is required for targeting Mtb to selective autophagy. However, removal of this danger sensor did not completely abrogate targeting. This parallels what we have seen when the DNA sensor cGAS is depleted; around 50% of Mtb bacilli are still targeted (21). There are several possible mechanisms that could explain this phenomenon. First, these two pathways may function in parallel, each targeting some fraction of Mtb bacilli, adding up to the total of ∼30% Mtb targeted in a wild-type cell. Future studies in cells lacking both cGAS/STING and galectin-8 could address this possibility. Second, it is possible that Rv1468c, Mtb’s ubiquitin-binding surface protein, contributes substantially to the ubiquitin cloud, and because of Rv1468c, removing host sensors will only ever decrease targeting to ∼15% (57). It is likely that some of the Rv1468c-bound ubiquitin chains serve as the substrates to recruit TAX1BP1 and other adapters, so using an Rv1468c mutant in future studies of host sensing pathways will help elucidate if additional host factors remain to be discovered in the targeting of Mtb. Ultimately, understanding the molecular mechanisms underpinning the host-pathogen interactions between Mtb proteins and macrophage proteins will be critical for understanding the innate immune response to Mtb.

Previous reports have observed galectin-3 and -8 colocalized with Mtb, and galectin-3 in particular is often used as a go-to marker of membrane damage during bacterial infection (18, 33–38). However, our results indicate that in macrophages infected with Mtb, galectin-8 may be a more suitable marker for membrane damage since in both macrophage cells lines and primary macrophages, galectin-8 recognizes two to three times more damaged phagosomes than galectin-3. This suggests that the galectin-3^+^ population of Mtb may significantly underestimate the true number of damaged phagosomes and cytosolically exposed bacilli. While our measurements are difficult to directly compare with other published reports—especially since we focused on earlier time points (6 h) rather than later time points (1 to 6 days)—our results are consistent with the existing literature, as we see comparable portions of galectin-3^+^ Mtb: roughly 10 to 20% depending on the study, conditions, time point, mycobacterial species, and method of quantification (18, 33, 34, 38).

Previous studies of galectins and Mtb have also examined the *in vivo* requirement for individual galectins during infection. Interestingly, these studies found that galectin-8^−/−^ and galectin-3^−/−^ mice succumb more rapidly to Mtb infection, suggesting that these galectins are required for controlling Mtb infection (22, 60). However, these studies did not further interrogate how galectins contributed to innate immunity during Mtb infection, and galectins are multifunctional proteins that play a multitude of roles *in vivo* beyond their intracellular function in macrophages. To understand how individual galectins contribute to macrophages’ ability to control Mtb *in vivo*, future studies will need to interrogate innate immune time points/readouts during *in vivo* infection, and infecting mouse models with macrophage-specific deletion or overexpression of galectins could be especially illuminating. Such experiments would provide some of the best evidence to date of how selective autophagy in particular contributes to the control of Mtb infection *in vivo*. A previous study infected p62^−/−^ mice with Mtb but found no differences between wild-type and p62^−/−^ mice (25); however, as demonstrated here and elsewhere, several selective autophagy adapters are involved in detecting and targeting Mtb, so it is likely that removing multiple adapters will be necessary to study the *in vivo* requirement of selective autophagy adapters.

Finally, the finding that overexpression of galectins can enhance macrophages’ ability to control Mtb is particularly noteworthy. Several host and bacterial factors can be mutated to diminish the targeting of Mtb to selective autophagy, but there are few known ways to enhance this targeting (61). For many intracellular bacterial pathogens, including Mtb, the targeted percentage is rarely above ∼30%, suggesting this might be a biological setpoint that is difficult to overcome. However, it seems that galectin overexpression, even at the moderate levels permitted by our lentiviral expression system (see Fig. S1B), is able to accomplish just that. Identifying a class of proteins like galectins that can enhance targeting without causing significant off-target effects is extremely valuable in our efforts to develop future anti-TB therapies. For instance, overexpression or stimulation of cGAS, which is required for selective autophagy targeting, may enhance the number of targeted Mtb bacilli, but chronic activation of cGAS also results in enhanced production of type I IFNs, which are probacterial and cause increased disease pathology *in vivo* (13, 15, 62–64). While chronic overexpression of galectins can have detrimental effects (65), using small molecules to augment the function of galectins, specifically during infection, might be an especially attractive strategy for the future development of host-directed TB therapies.

## MATERIALS AND METHODS

### Cell lines and cell culture.

RAW 264.7 cells (ATCC TIB-71) and HEK293T cells (ATCC CRL-3216) were cultured in Dulbecco modified Eagle medium (DMEM) plus 10% heat-inactivated fetal bovine serum (FBS) plus 20 mM HEPES at 37°C with 5% CO_2_. U937 cells (ATCC CRL-1593.2) were cultured in RPMI plus 10% heat-inactivated FBS plus 20 mM HEPES and differentiated with 100 ng/ml phorbol myristate acetate (PMA) for 48 h prior to infection. Lenti-X (TaKaRa Bio) cells were used to produce lentiviral particles. Where necessary, RAW 264.7 cells were selected with and maintained in 5 μg/ml puromycin (InvivoGen) or 5 μg/ml blasticidin (InvivoGen) and U937s in 1 μg/ml puromycin. For infections, antibiotics were omitted from culture media. RAW 264.7 cells were plated at 2 × 10^5^ cells/well in on circular glass coverslips in 24-well tissue culture (TC) dishes for immunofluorescence experiments and at 3 × 10^5^ cells/well in 12-well TC dishes for luciferase growth assays.

Tagged expression constructs were made by first cloning cDNAs from RAW 264.7 or U937 RNA into pENTR1a entry vectors modified to contain in-frame epitope or fluorescent tags (Addgene, plasmid 17396) (21, 43, 66, 67). Constructs were fully Sanger sequenced (Eton Biosciences, San Diego, CA) to verify the tagged proteins were complete, in-frame, and error-free. Constructs were then Gateway cloned with LR Clonase (Invitrogen) into pLenti destination vectors (Addgene, plasmid 19067) (66). Expression of tagged proteins was confirmed by transfecting HEK293Ts with 1 μg of pDEST and harvesting cell lysates after 1 to 2 days of expression. Proteins were separated by SDS-PAGE and visualized by Western blotting with primary antibodies for FLAG (clone M2; Sigma-Aldrich, catalog no. F1804) and HA (Roche, catalog no. 11867423001).

To make RAW 264.7 and U937 stable expression cells lines, Lenti-X 293T cells (TaKaRa Bio) were cotransfected with pLenti plasmids and the packaging plasmids psPAX2 and pMD2G/VSV-G (Addgene, plasmids 12259 and 12260) to produce lentiviral particles. Macrophage cell lines were transduced with lentivirus for two consecutive days plus 1:1,000 Lipofectamine 2000 (Invitrogen) and selected for 3 to 5 days with antibiotic. Expression of tagged proteins was confirmed by Western blotting with antibodies against corresponding epitope tags.

Bone marrow-derived macrophages were generated as previously described (21, 68). Briefly, femurs and tibias from wild-type C57BL/6J mice (housed, bred, and studied at Texas A&M Health Science Center under approved Institutional Care and Use Committee guidelines) were flushed to collect bone marrow and then plated at 1 × 10^7^ cells/plate (15-cm non-TC plates) in 20 ml of BMDM media (DMEM with glutamine, 1 mM sodium pyruvate, 20% FBS, 10% 3T3 MCSF [macrophage colony-stimulating factor]). Next, 30 ml of fresh medium was added after 3 days, and cells were collected and frozen after 6 to 7 days. BMDMs stably expressing GFP-tagged galectins were generated as previously described (69). Briefly, lentivirus was produced as described above. Bone marrow was harvested from wild-type mice and plated at 1.5 × 10^7^ cells/plate (10-cm non-TC plates) in 10 ml of BMDM media plus additional 10% 3T3 MCSF. After 2 days, cells were transduced with 6.5 ml of lentivirus plus 1:1,000 Lipofectamine for two consecutive days. After 1 day of recovery in BMDM media plus 10% 3T3 MCSF, cells were selected with 2.5 μg/ml puromycin for 3 to 5 days, when BMDMs were efficiently selected and fully differentiated. GFP and GFP-galectin expression was monitored over the course of transduction and selection using fluorescence microscopy.

Galectin-8 was knocked down in BMDMs using transient transfection of small interfering RNAs (siRNAs) as previously described (43, 67). Silencer Select siRNAs were purchased from Invitrogen (negative control 1; Lgals8–s80031). Differentiated BMDMs were thawed and plated in 6-well dishes at 6 × 10^5^ cells/well. The next day, each well of BMDMs was transfected with 20 μl of 2.75 μM negative control or galectin-8-targeting siRNA using Viromer Blue (OriGene) according to the manufacturer’s instructions. The next day, BMDMs were plated for infections; a fraction of the transfected BMDMs was collected in TRIzol at the time of infection to measure the knockdown efficiency.

### Bacterial infections.

Erdman was used as the parental Mtb strain for these studies (15, 17, 21). Our Erdman strain is highly virulent, as measured by both *in vivo* mouse infections and *ex vivo* macrophage infections (43, 68). The wild-type mCherry, Δ*esat-6* mCherry, and luxBCADE strains have been described previously (17, 21, 41–43). Mtb cultures were grown in Middlebrook 7H9 (BD Biosciences), 10% BBL Middlebrook OADC (Becton, Dickinson), 0.5% glycerol, and 0.1% Tween 80 at 37°C in roller bottles. Strains were propagated with minimal passage to preserve their virulence.

Mtb infections were performed as previously described (15, 21, 43). Briefly, cultures grown to 0.6 to an optical density at 600 nm (OD_600_) of 0.8 were spun at 500 × *g* for 5 min to remove large clumps and then spun again at 3,000 × *g* for 5 min to pellet bacteria. After two washes with PBS, bacteria were resuspended in PBS, sonicated briefly to disrupt clumps, and then spun once more at 500 × *g* for 5 min to remove remaining clumps. The OD_600_ of the bacterial suspension was used to calculate the volume needed for the desired multiplicity of infection (MOI) of 1 (1 OD = 3 × 10^8^ bacteria/ml). Bacteria were diluted in DMEM–10% horse serum and added to cells. Infections were synchronized by spinning for 10 min at 1,000 × *g*, and cells were washed twice with PBS and cultured in regular media. When experiments lasted for more than 24 h, cell culture medium was replaced daily.

For IF experiments, at the indicated time points, coverslips were transferred to 4% fresh paraformaldehyde in PBS, fixed for 20 min, and washed three times with PBS. For luciferase experiments, cells were washed twice with PBS, lysed in 0.5% Triton X-100, and transferred to a white luminescence plate (LumiTrac 96-well plates; Greiner Bio-One). Luminescence was measured using a Tecan Infinite 200 Pro. For CFU experiments, infected cells were lysed in 0.5% TritonX-100 and serially diluted in PBS + 0.1% Tween 80. Dilutions were spread on 7H10 plates (supplemented with 10% OADC [oleic acid-albumin-dextrose-catalase] and 0.5% glycerol) and grown at 37°C for 3 to 4 weeks before enumeration of the colonies. For both luciferase and CFU 0-h time points, cells were lysed after PBS washes rather than being returned to cell culture media.

An LDH assay to measure cell death was performed using an LDH-Glo cytotoxicity assay (Promega) according to the manufacturer’s instructions. Briefly, at each time point, 5 μl of culture supernatant from every well of infected cells (performed in triplicate or quadruplicate) was diluted 1:200 in 1 ml of LDH storage buffer (200 mM Tris [pH 7.4], 10% glycerol, 1% BSA). Samples were immediately assayed in duplicate by mixing 50 μl of premixed enzyme/substrate and 50 μl of diluted supernatant in a 96-well LumiTrac plate. Luminescence was read every 30 min for 4 h, and values collected at 2 h (when values were in the linear detection range) were used to compare cell lines. To permit accumulation of LDH over the course of infection, fresh medium was added daily to cells rather than changing media daily.

Listeria monocytogenes infections were also performed as previously described (21). RAW 264.7 cells were plated at 1 × 10^8^ cells per plate in 10-cm dishes. Listeria monocytogenes Δ*actA* (parental strain 10403, a gift from Dan Portnoy) was grown in brain heart infusion (BHI; BD) at 30°C overnight without shaking. Culture was diluted 1:10 in BHI and grown for 3 to 4 h at 37°C without shaking until it reached an OD_600_ of ∼0.6. Bacteria were washed twice with Hanks balanced salt solution (HBSS), and the OD of the resulting bacterial suspension was used to calculate the volume needed for an MOI of 5 (1 OD = 1 × 10^8^ bacteria/ml). Bacteria were diluted in HBSS and added to cells. After incubation of the cells and bacteria for 30 min at 37°C, the cells were washed twice with HBSS plus 40 μg/ml gentamicin and then cultured in media plus 10 μg/ml gentamicin until harvest.

### CRISPR/Cas9 knockouts.

RAW 264.7 cells stably expressing FL-Cas9 were generated by transducing RAW 264.7 cells with lentivirus containing LentiCas9-BLAST (Addgene, plasmid 52962) (70). These cells were selected with 5 μg/ml blasticidin (Invivogen) for 3 to 5 days and then with 10 μg/ml blasticidin for an additional 1 to 2 days. FL-Cas9 expression was confirmed by Western blot analysis.

sgRNAs for each galectin gene were designed using the sgRNA Designer using the CRISPRko website (https://portals.broadinstitute.org/gpp/public/analysis-tools/sgrna-design) and synthesized by IDT (71, 72). sgRNAs used for each galectin were as follows: gfp-1, GGGCGAGGAGCTGTTCACCG; gfp-2, CAGGGTCAGCTTGCCGTAGG; gal3-1, TCTGGAAACCCAAACCCTCA; gal3-2, GGCTGGTTCCCCCATGCACC; gal8-1, TCAGTAATGGTGCCAACATA; gal8-2, CAGTAATGGTGCCAACATAG; gal9-1, TACCCTCCTTCCTCAAACCG; and gal9-2, ACCCCCGGTTTGAGGAAGGA. Primers were cloned into LentiGuide-Puro (Addgene, plasmid 52963) by phosphorylating, annealing, and ligating primers into digested vector (70, 73). sgRNA plasmids were validated by Sanger sequencing using the universal pLKO.1/hU6 promoter primer (Eton Biosciences, San Diego, CA). Lentivirus with sgRNAs were produced and used to transduce low-passage-number FL-Cas9 RAW 264.7 cells. After selection with 5 μg/ml puromycin, the knockout efficiency was assessed at the population level. Using cells from the two most efficient sgRNAs, individual cells were serially diluted and plated into 96-well dishes to isolate clonal populations. When clones grew, populations were expanded, and each sample was assayed for mutations by amplifying a 500-bp segment of genomic DNA around the mutation. These PCR fragments were Sanger sequenced using nested primers and compared to controls using TIDE analysis. Clones with homozygous nonsense mutations were further validated by measuring galectin RNA expression.

Triple-knockout lines were made using a modified multiplexed lentiviral sgRNA system (74). The Cas9 in the lentiviral plasmid from this system was replaced with the puromycin resistance gene from a pDEST plasmid, which allowed for drug selection of a sgRNA array in RAW 264.7 cells already expressing FL-Cas9. sgRNAs for individual galectin genes were cloned into the sgRNA expression plasmids and assembled via Golden Gate assembly into the lentiviral backbone as previous published (74). RAW 264.7 cells expressing FL-Cas9 were transduced with lentivirus containing sgRNA arrays (GFP sgRNAs or galectin sgRNAs), and cells were selected, cloned, and screened as described above.

### Immunofluorescence.

Coverslips with fixed cells were blocked and permeabilized in 5% nonfat milk in PBS + 0.1% saponin for 30 min. Coverslips were then stained with primary antibody diluted in PBS with 5% milk and 0.1% saponin for 2 to 4 h. Primary antibodies used in this study were FLAG (Clone M2, Sigma-Aldrich, F1804; 1:1000), FLAG (Sigma-Aldrich, F7425; 1:1,000), HA (Roche, 11867423001; 1:1000), LC3 (Invitrogen, L10382; 1:250), ubiquitin (clone FK2; Millipore Sigma, 04-263; 1:500), p62 (Bethyl, A302-855A; 1:500), p62 (Abcam, ab56416, 1:500), TAX1BP1 (Bethyl, A303-791A; 1:500), OPTN (Bethyl, A301-829A; 1:500), and GFP (Abcam, ab183734; 1:500). Coverslips were washed three times in PBS and stained with secondary antibodies (goat anti-rabbit Alexa Fluor 488, goat anti-rat Alexa Fluor 647, and/or goat anti-mouse Alexa Fluor 647; Invitrogen, 1:1,000) and DAPI (1:10,000) in PBS–5% milk–0.1% saponin for 1 to 2 h. Coverslips were then washed twice with PBS and twice with water and mounted using Prolong Gold Antifade Mountant (Thermo Fisher). Cells were imaged on an Olympus Fluoview FV3000 confocal laser scanning microscope. Three coverslips per genotype were imaged, and at least 300 bacteria per coverslip were assessed and counted.

### Immunoprecipitations.

HEK293T cells were plated at 5 × 10^7^ cells per plate in 6-cm TC dishes. The following day, cells were transfected with 2 to 5 μg of indicated expression plasmids using PolyJet (SignaGen) according to the manufacturer’s instructions. Typically, 1 μg of bait plasmid and 1 to 4 μg of prey plasmid were cotransfected. After 2 days, cells were washed with PBS, lifted using PBS-EDTA, and pelleted by centrifuging at 3,000 × *g* for 5 min. Cells were lysed in lysis buffer (150 mM Tris [pH 7.5], 50 mM NaCl, 1 mM EDTA, 0.075% NP-40, protease inhibitors), and lysates were cleared of cellular debris and nuclei by spinning at 7,000 × *g* for 10 min. Then, 5% of the cleared lysate was saved as the “whole-cell lysate,” mixed with 4× Laemmli sample buffer with fresh β-mercaptoethanol (Bio-Rad), and boiled for 5 min. The remaining cell lysate was incubated with prewashed (three times in 1 ml of lysis buffer) 20 μl of antibody-conjugated beads/resin (FLAG [EZview Red anti-FLAG M2 affinity gel, Sigma-Aldrich]; HA [Pierce anti-HA agarose, Thermo Scientific]) for 30 to 60 min at 4°C with rotation. Beads were washed three times with 1 ml of wash buffer (150 mM Tris [pH 7.5], 50 mM NaCl, 1 mM EDTA, 0.05% NP-40), and proteins were eluted with an excess of FLAG peptide (Sigma) or HA peptide (Sigma) resuspended in lysis buffer plus 1% NP-40. Eluates were mixed with 4× sample buffer and boiled for 5 min. Proteins in whole-cell lysates and immunoprecipitations were resolved by SDS-PAGE and imaged by Western blotting with FLAG or HA antibodies (1:5,000 in Li-Cor TBS blocking buffer), corresponding Li-Cor secondary antibodies (1:15,000), and a Li-Cor Odyssey Fc imager. Immunoprecipitations in RAW 264.7 cells stably expressing 3×FLAG-tagged proteins were performed using the same protocol and workflow using cells infected with Listeria monocytogenes 1 or 2 h postinfection.

### RNA extraction and RT-qPCR.

Cells were harvested in TRIzol and RNA was extracted using Direct-Zol RNA MiniPrep kits (Zymo Research) with at least 1 h of on-column DNase treatment. cDNA was made using iScrpit (Bio-Rad), and gene expression was quantified using relative standard curves on a QuantStudio 6 Flex (Applied Biosystems) with PowerUp SYBR green Master Mix (Applied Biosystems). Primers for *Actb* (F-GGTGTGATGGTGGGAATGG and R-GCCCTCGTCACCCACATAGGA), *Lgals3* (F-CTGGAAACCCAAACCCTCAA and R-AGGAGCTTGTCCTGGGTAG), *Lgals8* (F-CCCTATGTTGGCACCATTACT and R-GCTGAAAGTCAACCTGGAATCT), and *Lgals9* (F-GCCCAGTCTCCATACATTAACC and R-GTTCTGAAAGTTCACCACAAACC) were synthesized by IDT.

### Exosomes.

RAW 264.7 cells were plated at 5 × 10^7^ cells per plate in 10 cm dishes. After 1 or 2 days in culture, cell culture medium was collected, and cells were washed once with PBS and harvested by scraping. For whole-cell lysates (WCLs), cells were pelleted and lysed directly in 1× Laemmli sample buffer with fresh β-mercaptoethanol (Bio-Rad), sonicated to break up DNA, and boiled for 5 min. Culture medium was precleared of dead cells and cell debris by spinning for 5 min at 3,000 × *g*. Exosomes were then collected by ultracentrifugation for 1 h at 100,000 × *g*. Exosome pellets were resuspended directly in 1× sample buffer and boiled for 5 min. Proteins from WCLs and exosomes were resolved and imaged by SDS-PAGE and Western blot analysis as described above using antibodies for FLAG (Sigma, F-1804; 1:5,000), Alix (Abcam, ab117600; 1:2,500), and syntenin-1 (Abcam, ab19903; 1:2,500).

### Data analysis and presentation.

Statistical analysis was performed using Prism (GraphPad) with Student unpaired two-tailed *t* tests. Graphs were generated with Prism, figures were generated with Adobe Illustrator and Photoshop, and diagrams and schematics were generated with BioRender.com as indicated in figure legends. At least three independent experiments were performed, and data presented is representative of these experiments. Figure legends indicate whether error bars indicate standard deviations (SD) or standard errors of the mean (SEM).
