# Supplementary figures and images for: Galectin-8 Senses Phagosomal Damage and Recruits Selective Autophagy Adapter TAX1BP1 To Control Mycobacterium tuberculosis Infection in Macrophages
Source: mBio. 2021 Jul 6;12(4):e01871-20. doi: 10.1128/mBio.01871-20 (PMC8406326; doi:10.1128/mBio.01871-20)

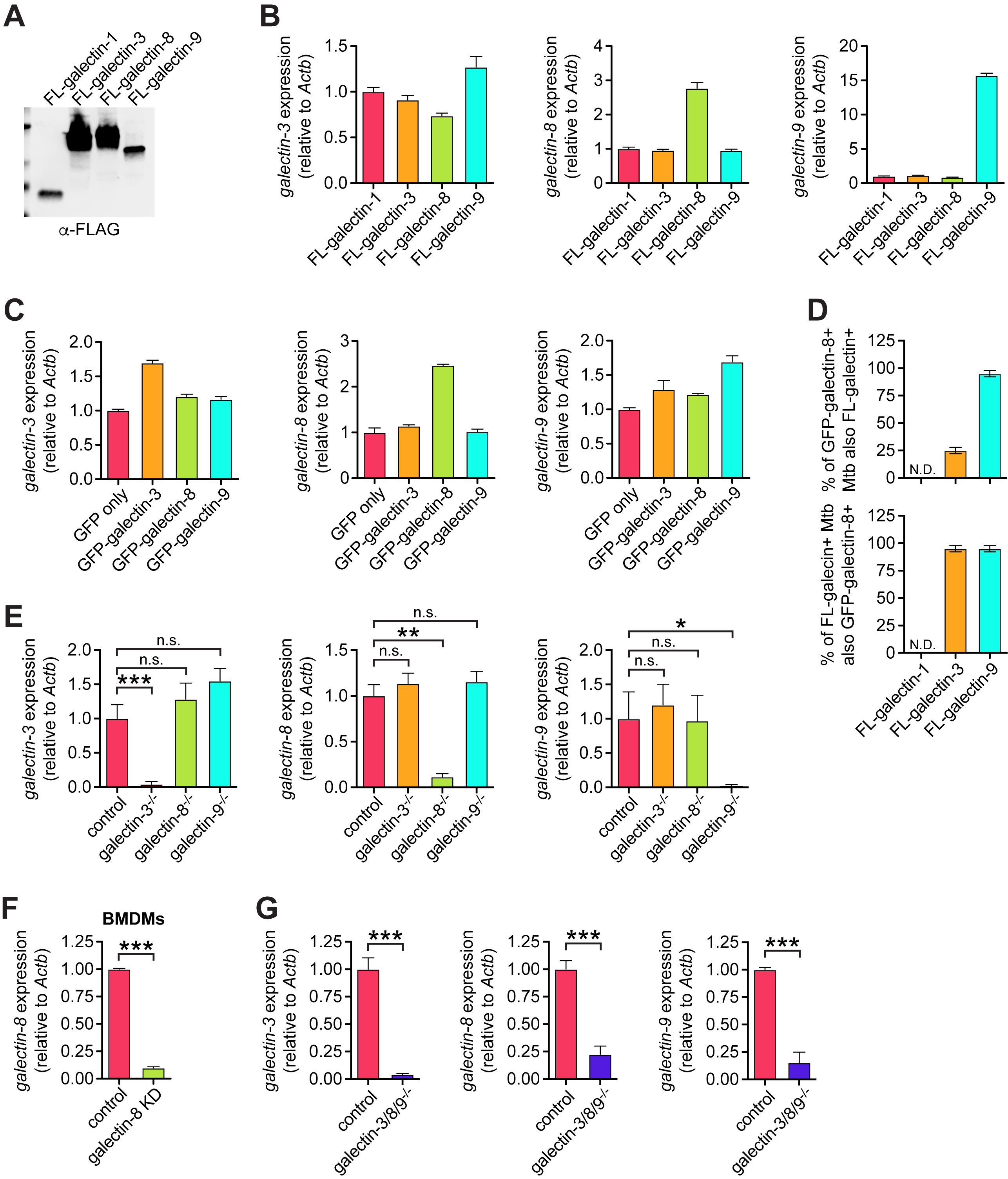

Supplement: FIG S1 [file mbio.01871-20-sf001.tif]

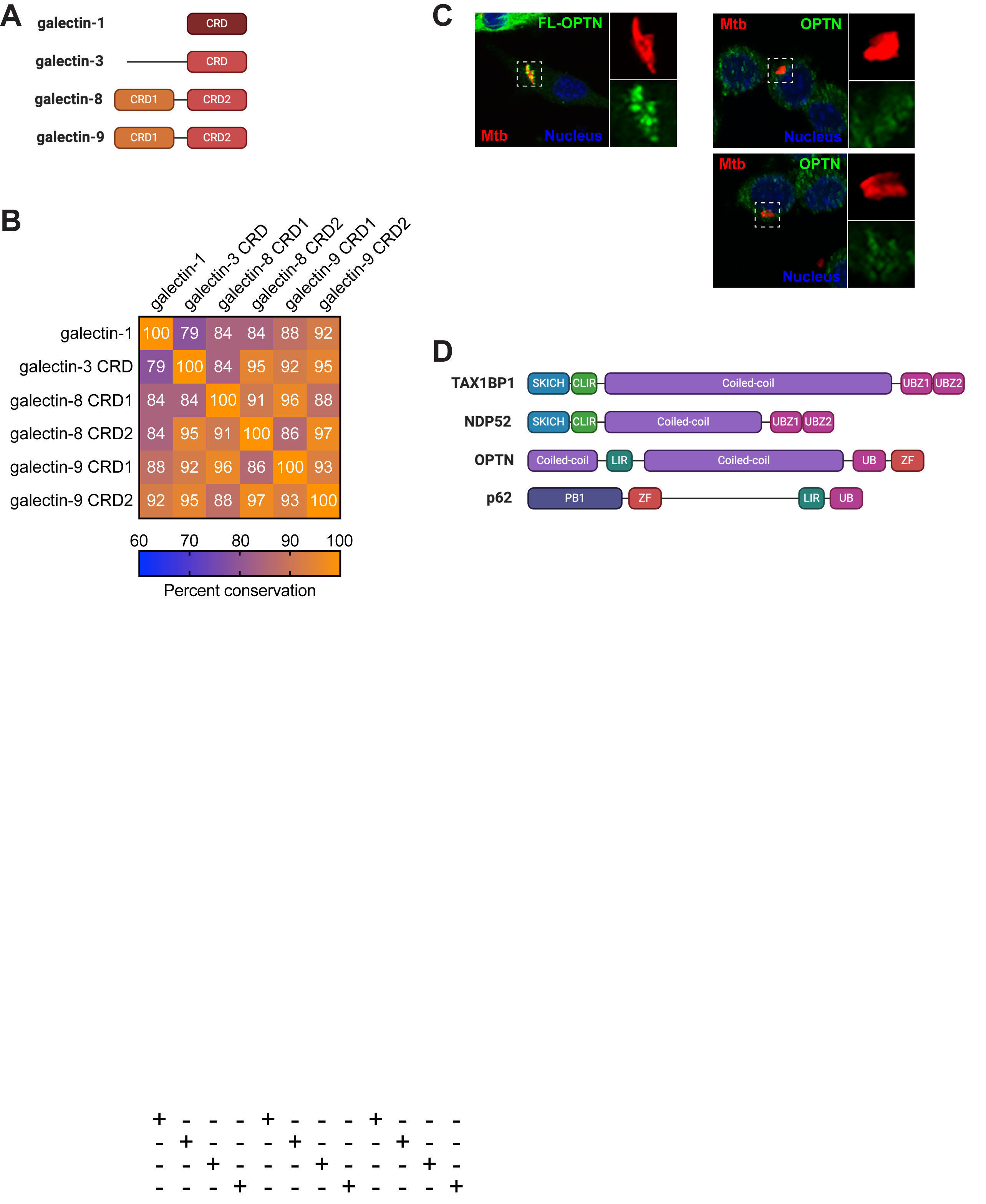

Supplement: FIG S2 [file mbio.01871-20-sf002.tif]
